# Analysis of Thrombolysis Process for Acute Ischemic Stroke in Urban and Rural Hospitals in Nova Scotia Canada

**DOI:** 10.3389/fneur.2021.645228

**Published:** 2021-03-15

**Authors:** Tessa Bulmer, David Volders, Noreen Kamal

**Affiliations:** ^1^Department of Industrial Engineering, Faculty of Engineering, Dalhousie University, Halifax, NS, Canada; ^2^Interventional & Diagnostic Neuroradiology, QEII Health Sciences Centre, Nova Scotia Health, Halifax, NS, Canada; ^3^Department of Radiology, Faculty of Medicine, Dalhousie University, Halifax, NS, Canada

**Keywords:** acute ischemic stroke, tissue plasminogen activator (tPA), thrombolysis, door-to-needle (DTN) time, Emergency Department (ED), delay factors, stroke pathways, urban-rural treatment gap

## Abstract

**Background:** Stroke is a devastating disease, but it is treatable with alteplase or tissue plasminogen activator (tPA). The effectiveness of tPA is highly time-dependent, meaning rapid treatment is critical. Fast treatment with tPA has been reported in many urban hospitals, but hospitals in rural locations struggle to reduce treatment times. This qualitative study examines current thrombolysis processes in one urban and two rural hospitals in Nova Scotia, Canada, by mapping and comparing the treatment process in these settings for acute ischemic stroke (AIS) patients, and by analyzing the healthcare professionals views on various treatment topics.

**Methods:** Structured interviews were conducted with healthcare professionals involved in stroke treatment across the three sites. The interviews focused on the various activities in the thrombolysis treatment at each site. Additionally, participants were asked about the following 10 topics: comfort treating acute ischemic stroke patients; perceptions about tPA; appropriate tPA treatment window; stroke patient priority; tPA availability; patient consent; urban-rural treatment differences; efficiency of their treatment process; treatment delays; and suggested process improvements. Results were analyzed using the Framework Method, as well as through the development of process maps.

**Results:** Twenty three healthcare professionals were interviewed at 2 rural hospitals and 1 urban hospital. Acute ischemic stroke patients are triaged as the highest or urgent priority at each included site. Physicians are more hesitant to treat with tPA in rural settings. A total of 11 urban-rural treatment differences were noted by the rural sites. Additionally, 11 patient-related and 29 system treatment delays were described. A process map was developed for each site, representing the arrival by ambulance and by private vehicle pathways.

**Conclusions:** Guidelines and clear protocols are critical in reducing treatment times and ensuring consistent access to treatment. The majority of treatment delays encountered are system delays, which can be appropriately planned for to reduce delays within the care pathway. There is a general consensus that there is an urban-rural treatment gap for acute ischemic stroke patients in Nova Scotia, and that continuing education is key in rural hospitals to improve Emergency Department (ED) physician comfort with treating patients with tPA.

## Introduction

In 2019, stroke ranked fourth for cause of death in Canada (1) and is the leading cause of severe disability (2). When having a stroke, a person loses ~1.9 million neurons, and 14 billion synapses every minute (3). Additionally, each hour without treatment results in a person losing the amount of neurons that would normally take 3.6 years to be lost (3), leading to the popularly referenced motto “time is brain.” Treatment with alteplase or tissue plasminogen activator (tPA) for Acute Ischemic Stroke (AIS) has been a proven treatment since the mid 1990's (4). Patients can benefit from being treated with tPA up to 4.5 h from time of symptom onset (5). Although, it was shown that patients have better outcomes when treated more rapidly with tPA (5, 6), as the effectiveness of tPA is highly time dependent (7). For maximal benefit, patients should be treated with tPA as quickly as possible (5, 6). Therefore, current Canadian guidelines indicate that patients should be treated with tPA within 30 min from their arrival at the hospital (8). This is referenced as a door-to-needle (DTN) time, meaning the length of time it takes from the moment they arrive at the hospital to the start of tPA treatment.

Delays in tPA treatment have been found to be associated with patient factors as well as system factors (9). To overcome these delay factors, there has been extensive research done on the processes and strategies that result in shorter DTN times (10, 11). It has been shown that using multiple strategies can reduce a hospital's median DTN time to 20 min (12). The top strategies include: receiving pre-notification by Emergency Medical Services (EMS) and having a single call activation of the stroke team; moving the patients directly to the computed tomography (CT) scanner on the EMS stretcher; having a rapid registration process; and administering tPA in the scanner area (10). Acute stroke management practices in rural areas have been labeled as sub-optimal, resulting in a gap in quality of treatment in urban and rural areas (13). A recent study that reduced DTN times across an entire population showed that urban and community hospitals with access to neurologists at all times were able to reduce their median DTN times to 35 and 34 min from 65 to 73 min, respectively, but rural hospitals were only able to reduce their DTN times to a median of 54 min from 84 min (14). There is a need to better understand the barriers to fast treatment in rural hospitals to ensure equitable care.

It was anticipated that the disparity between rural and urban treatment of AIS is pronounced in Nova Scotia, a Canadian province located on the east coast of the country, due to resource differences and population distribution. Stroke treatment in Nova Scotia is challenging as small populations are dispersed over wide rural areas. According to the 2016 Canadian Census, 43% of the province's population resides in rural communities (15). It was shown that there is a higher risk of incident stroke in rural areas (16), estimated to be 23% higher in large rural areas and 30% higher in small rural towns, when being compared with urban areas (16). Therefore, there is a critical need to ensure that rural populations in Nova Scotia receive equitable access to efficient treatment. This study aims to shed some light on the differences in the thrombolysis process between urban and rural hospitals in Nova Scotia. The objectives for the study are the following: (1) to analyze the healthcare professionals views on various treatment topics; and (2) to map and compare the thrombolysis treatment process for acute stroke patients in urban and rural settings in Nova Scotia.

## Methods

A qualitative study was conducted in order to better understand the treatment process in rural and urban hospitals. There were two rural sites chosen and one urban site to be able to compare the differences between urban and rural hospitals. The consolidated criteria for reporting qualitative research (COREQ) was consulted to report key aspects of the study in the following domains: the research team, study design, and analysis and findings (17). The qualitative study was completed by conducting interviews with key healthcare professionals who are involved in AIS treatment at each site. TB conducted the interviews and data analysis, and was a Master of Applied Science in Industrial Engineering student at the time of the study.

### Site Context

These sites were selected to provide a fair representation, with a limited sample size, of the different hospitals in Nova Scotia providing thrombolysis treatment, based on the presence and absence of neurologists in-hospital, and varying CT technologist availability. Site 1 is the urban site and the province's only comprehensive stroke center. Site 1 is a large teaching hospital with ~800 beds, and is a level 1 trauma center. Some important distinctions regarding this site are that it is located in an urban setting, neurologists are always available during the treatment process, CT technologists are always available within the hospital; and it is the only hospital in Nova Scotia to provide endovascular treatment (EVT) to AIS patients. Site 2 is a rural hospital with ~200 beds located in a small town. A distinction from the urban site (Site 1) is that ED physicians are primarily responsible for thrombolysis, and they do not have neurologists on site. They do however similarly have a CT technologist always available within the hospital. Site 3 is another rural hospital with roughly 100 beds, located in a small town, with ED physicians primarily responsible for thrombolysis. This site does not have access to CT technologists out of hours within the hospital. Out of hours, the on-call CT technologist has to travel into the hospital when required. The three sites defined regular hours as 8 am to 4 pm or 5 pm, Mondays to Fridays. Out of hours are considered all times outside of regular hours, meaning evenings and weekends. Human resource differences and the resulting treatment process changes, between regular and out of hours, are further defined for each site in the treatment process results section. The described site distinctions and the local target and current median DTN times for each site are shown in Table 1. It can be seen that Site 2 currently has the lowest median DTN time, while Site 3 has the highest.

**Table 1:** Site distinctions with local target and current median door-to-needle times.

**Site**	**Site Distinction**	**Local Target Median DTN Time (min)**	**Current Median DTN Time (min) (18) June 2019 to May 2020**
Site 1 (Urban)	• Province's only comprehensive stroke center • Large urban teaching hospital • Level 1 trauma centre • Approx. 800 beds • Neurologists and stroke neurologists • 24/7 CT technologists within hospital	30	50
Site 2 (Rural)	• Rural hospital • Approx. 200 beds • ED physicians • 24/7 CT technologists within hospital	30	40
Site 3 (Rural)	• Rural hospital • Approx. 100 beds • ED physicians • CT technologists within hospital during regular hours, and some evenings/weekend shifts	60	77.5

*DTN, Door-to-Needle; CT, Computed Tomography; ED, Emergency Department*.

All of these sites have a stroke coordinator, who oversees the site's stroke care. Study participants were recruited for interviews from each site through the stroke coordinator. The participants include all of those involved in the treatment of acute ischemic stroke patients. In the urban setting, the target roles recruited included: ED nurse, ED physician, CT technologist, paramedic, neurologist, neurology resident, stroke neurologist, stroke nurse, and stroke coordinator. In the rural setting, the target roles that were recruited included: ED nurse, ED physician, CT technologist, paramedic, radiologist, and stroke coordinator.

### Ethics and Consent

This study was carried out in accordance with the recommendations of the Nova Scotia Health Research Ethics Board (REB). The study protocol, with the REB file number 1025975, was approved by the Board. Written informed consent was obtained for each participant involved in the study prior to the participant being interviewed. There was no compensation provided to any participants.

### Data Collection

Data was collected from September to October 2020, using structured interviews with various professionals that are involved in the AIS treatment process. The interview participants were selected by the site's stroke coordinator using a purposive sampling method. Upon selection, TB approached participants by email to complete the consent process and schedule their interview. Each interview included only TB and an individual participant, with no non-participants present. Participants completed a single interview, and were informed of the study objectives at the beginning of the interview.

Two interview guides were developed for the study and can be found in Supplementary Material. Pilot testing of the interview guides was completed with another student to determine comprehension of the questions, and interview duration. There was one standard interview guide for all healthcare professionals containing 33 questions, and one guide tailored to stroke coordinators containing 25 questions. Section 1 of the standard interview guide focuses on topics 1 to 10 listed in Table 2 to provide contextual details of the thrombolysis treatment at each site, while section 2 of the guide aimed to develop a detailed process map for the treatment process at each site from the perspective of different professionals. The questions for section 1 were developed based on the causes of delays that were identified in the literature (9) and the behavioral barriers to tPA treatment by emergency physicians (19–21). The questions for section 2 of the structured interview were developed for the purpose of understanding details of the specific steps in the treatment process from the perspective of the various healthcare professionals that were being interviewed. The basis of the questions for section 2 were based on previous studies that aimed to improve the tPA treatment process (10).

**Table 2:** 11 interview topics.

**Topic**
1. Comfort treating AIS patients
2. Perceptions about tPA
3. Appropriate tPA treatment window
4. AIS patient priority
5. tPA availability
6. Patient consent
7. Urban-rural treatment differences
8. Efficiency of their treatment process
9. Treatment delays at their site (patient-related and system)
10. Suggested process improvements at their site
11. Other topics

*AIS, Acute Ischemic Stroke; tPA, Tissue Plasminogen Activator*.

The stroke coordinator guide had many overlapping questions with the standard guide, although it did not include 3 of the topics from section 1: comfort treating AIS patients, perceptions about tPA, and patient consent as the stroke coordinators are not directly involved with AIS treatment. The interview guide prepared for stroke coordinators had additional questions in section 1 on the following topics: human resource limitations, key metrics in data analysis, following treatment protocols, as well as the opportunity to add any general background information regarding treatment at their hospital.

Interviews were designed to take ~45 min to complete and were conducted using Microsoft Teams (version 1.3.00.24758, Microsoft Corporation, Redmond, WA, USA), a video-conferencing platform, or via direct phone call. Audio recording was used to collect data for all interviews.

### Qualitative Analysis

The qualitative analysis of the interviews used the Framework Method, as well as process mapping for each included site. The Framework Method is a 7-step qualitative analysis technique used in health research that helps to reduce data into an organized matrix format that aids response comparison (22). The Framework Method provides high-level structured steps to the qualitative analysis process that can be applied to any topic, and allowed for study goals to be accomplished using a deductive approach. The 7 steps involved in the Framework Method are the following: (1) transcription of interviews: (2) familiarization with the interview content; (3) developing interview codes; (4) developing a working analytical framework by producing analysis categories: (5) applying the analytical framework: (6) charting data into the framework matrix: and (7) interpreting the data (22). Each interview was manually transcribed verbatim and was reviewed for familiarization of content. There was a single code associated with each question from the interview guide that directly related to the 10 pre-established topics that are listed in Table 2 and treatment process activities. Topic 11 noted in Table 2 encompasses other topics noted outside of the 10 pre-established topics from section 1. Due to the simplicity of the developed codes, it was not required for further categories to be established. The transcript content was coded accordingly, summarized data and meaningful quotes were entered into the framework matrices produced in Microsoft Excel, and the findings were interpreted. Member checking was done during the interview process to improve accuracy and validity of the results. Specifically, this was done by asking follow-up questions during the interviews or repeating back a participants' response to ensure clarity. For section 2 of the interview guide, email correspondence to some of the participants was conducted to obtain further details about their process.

To summarize, two matrices were produced for each of the three sites. No software was used to support analysis. The first matrix developed for each site included the 11 topics listed in Table 2. The second matrix consisted of process details extracted from interviews for each site for the purpose of developing process maps. Treatment process activities were ordered sequentially in the matrix using the thorough responses from each participant. The second matrix helped to clarify the sequence of activities at each site, as well as highlight site variation in the order of treatment process activities. The second matrix allowed for the sorting of the collected data and to highlight differences of the two pathways of focus: patients arriving by Emergency Medical Services (EMS) with pre-notification (for both regular and out of hours), as well as patients arriving by private vehicle (for both regular and out of hours). It was important to include the private vehicle pathway in addition to the EMS pathway as the beginning of the treatment processes differ. These two pathways were detailed graphically in a developed process map integrating both pathways for each site. Although EMS personnel were not included in the study, the pre-hospital pathway was felt to be effectively captured by interviewing healthcare professionals who closely interact with this portion of the care pathway. The pre-hospital pathway is critical to incorporate into site process maps as there are parallel activities that take place in-hospital during this period to prepare for the incoming AIS patient based on the information received from EMS personnel. Each process map produced in Microsoft Excel is considered a cross-functional workflow diagram, also referred to as a swim-lane diagram, as the diagram clearly sectioned the process activities into categories of people/departments who are responsible for sections of the process using grouping of activities and color coding to illustrate the care pathways (23).

## Results

There were 23 healthcare professionals recruited for the study. There were 8 participants from Site 1, and the roles included were: stroke coordinator, stroke neurologist, recent neurology resident graduate, current neurology resident, stroke nurse, ED nurse, and CT technologist. Site 2 included 9 participants, and Site 3 included 6 participants, each with the following roles involved in the interviews: stroke coordinator, ED physician, ED nurse, and CT technologist.

### Section 1 Interview Topic Results

The results from the topics 1–6, and 8 listed in Table 2, are summarized for each site in Table 3. It is important to note that patient priority is defined using the Canadian Triage and Acuity Scale (CTAS), which is 5 level acuity scale. Patients receiving a CTAS level of 1 are deemed to be the most urgent patients, while a CTAS 5 indicates the least urgency. The following lists some of the key topics with summarized descriptions of the results from these topics.

**Table 3:** Topics 1–6, and 8 summarized results.

**Topic**	**Site 1 (Urban)**	**Site 2 (Rural)**	**Site 3 (Rural)**
1. Comfort treating AIS patients (Low, Moderate, High)	High	High	Moderate
2. Perceptions about tPA (Hesitant, Neutral, Accepting)	Accepting	Neutral-Accepting	Hesitant-Accepting
3. Appropriate tPA treatment window (from onset of symptoms)	Ideally within 3 h, up to 4.5 h	Ideally within 3 h, up to 4.5 h	Ideally within 3 h or earlier, up to 4.5 h
4. AIS patient priority	CTAS 1, top priority	CTAS 2, urgent priority	CTAS 2, urgent priority
5. tPA availability	No issue	No issue	No issue
6. Patient consent	Given by patient or family member, but generally inferred consent	Given by patient or family member. Discussion on tPA risk factors	Given by patient or family member. Discussion on tPA risk factors, checklist completed
8. Efficiency of their treatment process	Efficient, but not optimal. Inefficiencies remain between imaging and administration stage	Efficient and streamlined. Some inefficiency remains in treatment decision stage	Efficient. Inefficiencies remain between imaging and administration stage

*AIS, Acute Ischemic Stroke; tPA, Tissue Plasminogen Activator; CTAS, Canadian Triage Acuity Scale*.

#### Comfort Treating AIS Patients and Perceptions About tPA

The healthcare professionals were generally comfortable treating AIS patients, noting how comfort can be fostered with higher treatment frequency, and that the time sensitive nature of the treatment is an obstacle. A recent graduate from the neurology residency program stated “over the years, we get quite a bit of comfort with treating patients with tPA and seeing stroke patients.” (Site 1 Participant 2). A Site 3 ED physician noted that the infrequency of providing thrombolytic treatment results in “not a good comfort” (Site 3 Participant 2) with the treatment, noting some ED physicians at Site 3 will not provide thrombolysis treatment and will instead draw on expertise of local internists, while their colleague added the pressure that is felt to provide this care pathway with a lack of individual comfort. An Emergency Department (ED) physician from Site 2 noted that the largest challenges are “the time pressure and the uncertainty of diagnosis” (Site 2 Participant 9) that accompanies AIS patients, which was echoed by another ED physician at the site. A Site 3 ED physician added that there are many stroke mimics that could mislead a patient's diagnosis, which leads to the feeling of uncertainty. The necessity to administer tPA rapidly was acknowledged by all healthcare professionals, one ED nurse stating “the faster the better” (Site 1 Participant 6), while another noted “time is of the essence” (Site 2 Participant 6).

In regard to the medication itself, some reservations were noted regarding the evidence and subsequent risks. A neurology resident at Site 1 saw tPA as a “powerful medication” adding that “most of the times the benefits greatly outweigh the risks of not giving the medication” (Site 1 Participant 3), but there is still a possibility of poor outcomes for patients. Physicians and nurses acknowledged there is a risk involved with treating patients with tPA, noting patients can develop bleeds post-administration. A Site 3 ED nurse expressed they hope the patients will recover from their deficit and experience an improved quality of life after being treated with tPA, and “that they don't have to suffer subsequent bad reactions” (Site 3 Participant 5). A stroke neurologist labeled tPA as an imperfect medication with a shortcoming due to there being “a fairly lengthy intravenous infusion required to give it” (Site 1 Participant 4) after giving the bolus dose. There was significant discussion by the physicians around the strength of the evidence for tPA, and based on their interpretation of the evidence, their comfort with treating with tPA. ED physicians from Site 2 and Site 3 stated that the evidence surrounding the use of tPA was lacking or unclear, one stating that “the evidence isn't super clear, but we still do it” (Site 2 Participant 2). Although, another ED physician at Site 2 did recognize “that the literature shows that it is an effective treatment” (Site 2 Participant 3), and others from Site 2 recognized that there were improvements among patients who received the drug.

#### Appropriate tPA Treatment Window (From Onset of Symptoms)

The majority of the healthcare professionals recognized that AIS patients could be treated up until 4.5 h from onset of symptoms, while generally it is preferred to treat within the first 3 h. A Site 3 ED physician expressed concern about an acceptable treatment window, specifically noting the ECASS III trial and the 3–4.5 h range, stating that “if we look at ECASS III in particular, I mean, they excluded a very significant group of people that are often the populations that we see in terms of age, previous stroke, diabetes, all that kind of stuff. So you get a little bit more nervous as you're going out.” (Site 3 Participant 3). The ECASS III trial (24), which extended the tPA treatment window from 3 to 4.5 h, excluded diabetic patients and those over 80 years of age. An ED physician from Site 2 also alluded to the trial, saying the 3 to 4.5 h period can be trickier, but still defined the treatment window to be up until 4.5 h. The same ED physician from Site 3 also noted concern about the timeline to treat patients with tPA safely, and referenced the NINDS trial while stating they feel the 0 to 90 min period seems the safest, and continued to say they most often tend to give thrombolytic treatment to younger patients. In contrast, participants from Site 1 and 2 noted that if a patient had severe deficits and the treatment window had passed, the team would consider providing the medication should it be appropriate to that particular case.

#### Patient Consent

There are varying opinions regarding patient consent for thrombolysis treatment, but it is clear that any consent process slows down the treatment process. It was noted that some physicians may want to have a lengthier discussion, some prefer a checklist to complete, and others believe no consent is required. A Site 3 ED physician detailed that the importance resides in the consent conversation being conducted without tainting the patient's perspective. They further went on to state “*I always tell patients that sometimes there's no right answer, that sometimes the disease will decide, but this is a treatment that we can offer you at this stage.”* (Site 3 Participant 3). Patients can understandably be distressed during the process, an ED physician from Site 2 describes “they're scared and we are sometimes throwing statistical numbers at them” (Site 2 Participant 9). If a patient is non-verbal or cognitively incapable to consent due to their current neurological deficit, a substitute decision-maker is included, which can be challenging to locate depending on the time of day. A stroke neurologist from Site 1 articulated the perspective of not requiring consent, stating that the patient is experiencing a neurological deficit “and it's an emergency situation, and so there is an argument that no consent is required.” (Site 1 Participant 4). The stroke neurologist described that it is challenging to expect someone who is experiencing a traumatic event directly, the patient, or indirectly as substitute decision-maker, to make a sound decision in that moment.

#### Urban-Rural Treatment Differences

Topic 7 involved asking participants if they felt there is a gap in AIS treatment between urban and rural hospitals in Nova Scotia. As the rural participants experience the challenges of the disparity of AIS treatment in the province, their responses were of focus, although all 3 sites acknowledged the existing urban-rural treatment differences. The rural participants noted the 11 instances summarized in Table 4. In Table 4 “Yes” indicates the site noted the difference, while “No” indicates the difference was not noted by that site. These indicators illustrate each site's perspective on the topic by defining which urban-rural treatment differences are noticed by each site. The instances include topics such as lack of access to further EVT treatment, healthcare professional expertise, resource availability, and distance of patients to the nearest hospital.

**Table 4:** Topic 7 summarized results noted by rural participants.

**Urban-rural treatment difference**	**Site 2 (Rural)**	**Site 3 (Rural)**
1. Rural patients do not locally have access to further EVT treatment	Yes	Yes
2. Urban site has neurologists, and additionally specialized stroke neurologists	Yes	Yes
3. Urban site has specialized neuroradiologists, while rural sites have radiologists	Yes	No
4. ED physicians are making treatment decisions in rural sites • Different level of expertise and comfort	No	Yes
5. Urban site has more human resources involved in treatment process • Not possible to have dedicated stroke team in rural setting	Yes	Yes
6. Urban site treatment process is more streamlined	Yes	No
7. Rural patients often live further from hospitals, affecting treatment window	Yes	Yes
8. Not one single standard of care, care provided differently in tertiary sites compared to rural sites	Yes	No
9. Many rural sites do not have CT scanners	Yes	Yes
10. Rural sites often do not have bloodwork results before tPA administration	Yes	No
11. EMS availability is reduced in rural areas • EMS covering larger geographical area	Yes	Yes

*EVT, Endovascular Thrombectomy; ED, Emergency Department; CT, Computed Tomography; tPA, Tissue Plasminogen Activator; EMS, Emergency Medical Services. “Yes” indicates the site noted the difference, while “No” indicates the difference was not noted by that site*.

An ED physician from Site 3 stated that there is a difference in the level of expertise and comfort due to the different volumes of strokes experienced at urban vs. rural sites. This sentiment leads to rural sites often consulting Neurology at Site 1, which rural participants noted could cause delay in the treatment process. However, Site 3 added that accessing a neurologist over the phone is generally quicker than consulting with Internal Medicine locally. There was also consensus that another factor was the amount of human resources available in the urban context, as well as the availability of EVT following thrombolysis, which is solely being offered at urban Site 1. Resources and equipment are a clear difference between the two settings. Both rural sites acknowledged that there is a difference due to the availability of CT scanners, as many community sites lack this equipment, which is critical to providing thrombolytic treatment. The two sites continued to discuss that EMS availability, the pre-hospital transport piece, is much more of a factor for their hospitals. They noted that EMS covers larger geographical areas in their settings, and thus cannot always respond to AIS patients as quickly as they would desire. Participants highlighted the severity of this challenge further as they discussed that rural patients often live further from hospitals, which further decreases the treatment window.

#### Treatment Delays at Their Site (Patient-Related and System)

Topic 9, treatment delays at each site, is categorized into 2 areas: patient-related delays, and system delays. As predicted, patient-related delays are delays that directly affect the patient. System delays are system factors associated with AIS tPA treatment, some directly affecting the patient and others affecting the process. Each site was asked to note the delays they felt were experienced at their respective site. The 11 patient-related and 29 system treatment delays noted by participants are shown in Tables 5, 6, respectively. In Tables 5, 6 “Yes” indicates the site noted they experienced that delay at their hospital, while “No” indicates the specified delay was not noted by that site. Once again, these indicators illustrate each site's perspective on the topic by stating which treatment delays are noticed by each site. It is clear that each site experiences more system treatment delays compared to patient-related delays. System treatment delays can be reduced with appropriate planning to minimize their impact on the care pathway. While patient-related treatment delays cannot be completely avoided, anticipating what delays could be encountered in this regard could also allow for this delay type to be minimized.

**Table 5:** Topic 9 (Patient-Related) summarized results noted by participants.

**Patient-Related Treatment Delay**	**Site 1 (Urban)**	**Site 2 (Rural)**	**Site 3 (Rural)**
1. Hypertension	Yes	Yes	Yes
2. Unclear time of onset	Yes	Yes	Yes
3. Patient is aphasic (obtaining consent)	Yes	Yes	Yes
4. Getting IV access (due to obesity or age of patient)	Yes	No	No
5. Patient requiring reversal of anticoagulation	Yes	Yes	No
6. Difficulty positioning patient in CT scanner	Yes	Yes	Yes
7. Patient is unstable	No	Yes	No
8. Fluctuating symptoms	No	Yes	Yes
9. Unclear story	No	No	Yes
10. Patient has comorbidities	No	No	Yes
11. Patient has another emergent medical condition	No	No	Yes

*IV, Intravenous; INR, International Normalized Ratio; CT, Computed Tomography. “Yes” indicates the site noted they experienced that patient-related delay at their hospital, while “No” indicates the specified delay was not noted by that site*.

**Table 6:** Topic 9 (System) summarized results noted by participants.

**System Treatment Delay**	**Site 1 (Urban)**	**Site 2 (Rural)**	**Site 3 (Rural)**
1. Treatment decision delay • Neurologist is slower to decide than stroke neurologist (Site 1) • Waiting for neurologist/stroke neurologist to arrive out of hours (Site 1)	Yes	Yes	Yes
2. Treatment decision consultation delay	No	Yes	Yes
3. Obtaining lab results	Yes	Yes	No
4. INR point-of-care machine does not always work	Yes	N/A	N/A
5. Inadequate staffing in ED	Yes	No	No
6. Getting IV access	Yes	Yes	No
7. Stroke recognition/diagnosis	Yes	Yes	Yes
8. Patient registration • Patient registration out of hours (Site 3)	Yes	No	Yes
9. Encountered occupied CT scanner	Yes	Yes	No
10. CT scanner not ready when patient arrived at imaging	Yes	No	No
11. Pre-hospital EMS transport delay	Yes	No	Yes
12. Other pre-hospital transport delays • Patient transfers from community hospitals (Site 2) • Patient transport into ED from private vehicle—ED staff not allowed to help bring patient into ED (Site 3)	No	Yes	Yes
13. Neurology initial assessment delay, assessment taking too long	Yes	N/A	N/A
14. Bloodwork collection before taking patient to scanner	Yes	No	No
15. Inadequate communication among healthcare professionals • No communication of patient's arrival at the ED (Site 1) • Not all information communicated through EMS patch (Site 2) • Miscommunication between radiologist and ED physician regarding which scans are being completed (Site 3) • No communication to imaging department of incoming AIS patient (Site 3)	Yes	Yes	Yes
16. Getting CT Report	No	Yes	No
17. Obtaining INR result	No	Yes	Yes
18. Getting patient history (none available)	No	Yes	No
19. Physical layout of hospital	No	Yes	No
20. Locating patient's next of kin	No	Yes	No
21. Inserting NG tube and Foley catheter before tPA administration	No	No	Yes
22. Determining patient's weight	No	No	Yes
23. Imaging delay due to lack of clarity regarding which patients require CTA scan completed	No	No	Yes
24. Interface with Radiology	No	No	Yes
25. Bloodwork collection out of hours	No	No	Yes
26. CT technologist having to travel to site out of hours	No	No	Yes
27. Radiologist reviewing images (if slow internet)	No	No	Yes
28. Receiving interpretation from radiologist	No	No	Yes
29. Challenge accessing visiting patient database	No	No	Yes

*INR, International Normalized Ratio; ED, Emergency Department; IV, Intravenous; CT, Computed Tomography; EMS, Emergency Medical Services; NG, Nasogastric; tPA, Tissue Plasminogen Activator; CTA, Computed Tomography Angiography. “Yes” indicates the site noted they experienced that system delay at their hospital, while “No” indicates the specified delay was not noted by that site*.

#### Suggested Process Improvements at Their Site

Topic 10 involved asking participants to state their thrombolysis process improvement suggestions for their site. The possible improvements noted by participants have been divided into pre-hospital and hospital-based suggestions shown in Table 7. The pre-hospital suggestions focus on EMS improvements such as method of communication and the information being conveyed, preparing the patient with two IVs, instructing the patient's next of kin, triage, and availability. The hospital-based suggestions focus on improvements in areas such as human and capital resources, the location for tPA administration, the sequence of activities in the care pathway, protocol adjustments, communication among healthcare professionals, and increasing continuing education.

**Table 7:** Topic 10 summarized results noted by participants.

	**Suggested Improvements**
	**Pre-hospital**
Site 1 (Urban)	• More direct information from paramedics (patient identity, clarity of problem, when possible more lead-time) • Modern secure telecom/video streaming systems for transmission of information of paramedic's assessment • EMS to put in two IVs before arriving to ED
Site 2 (Rural)	• EMS to automatically communicate patient identifiers • EMS getting patient's next of kin information and instructing them to go directly to hospital (or being easily accessible via their phone)
Site 3 (Rural)	• EMS triage • EMS availability
	**Hospital-based**
Site 1 (Urban)	• Stroke nurse available at all times (tPA administered in CT department) ° Alternatively, have an ED nurse travel with patient to imaging (tPA administered in CT department) ■ tPA stored within CT department in locked drug cupboard • Use INR point-of-care machine prior to imaging • Wait to collect blood sample until after imaging
Site 2 (Rural)	• Obtain INR point-of-care machine • Have patient's medication list automatically printed out (currently done by ED physician) • Bloodwork collected and 2nd IV put in on EMS stretcher before imaging • Obtain 2nd CT scanner • Improve communication among healthcare professionals • Increase emphasis on continuing education
Site 3 (Rural)	• Mix tPA and have treatment discussion while patient in scanner • Remove NG tube and Foley catheter requirement before tPA administration • Registration clerk and CT technologist in-hospital at all times • Clarification on which patients require CTA scan • Increase education piece to improve ED physician comfort with giving tPA

*EMS, Emergency Medical Services; IV, Intravenous; ED, Emergency Department; tPA, Tissue Plasminogen Activator; CT, Computed Tomography; INR, International Normalized Ratio; NG, Nasogastric; CTA, Computed Tomography Angiography*.

#### Other Topics

The analysis also allowed for data to be collected for other topics that do not fall under the 10 pre-established topics in section 1 of the interview guide, or the treatment process details in section 2, which has been defined as Topic 11, as shown in Table 2. The other topics noted are the following: human resource limitations, following protocols, quality of medical documentation and data collection obstacles, treatment speed phenomenon based on patient arrival time within the treatment window, opposing opinions toward thrombolysis between neurology and ED physicians, ability to determine efficacy of thrombolysis treatment in the ED, risks of patients traveling with lights and sirens with EMS on rural roads when unnecessary, and benefits of prioritizing stroke in the ED.

### Section 2 Treatment Process Results

**Figure 1:**
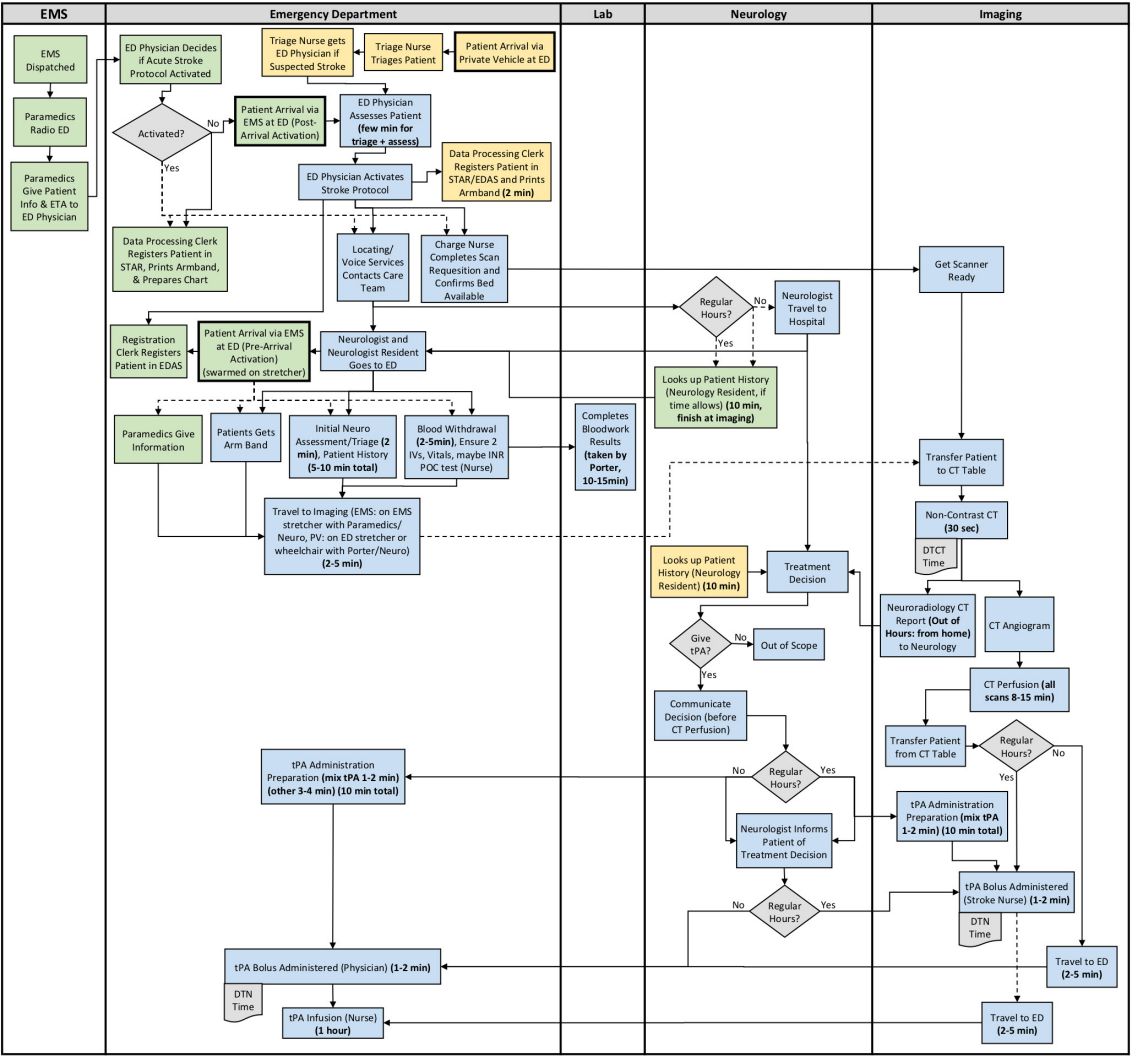
Site 1 (Urban) All Care Pathways Regular and Out of Hours Process Map. EMS, Emergency Medical Services; PV, Private Vehicle; ED, Emergency Department; CT, Computed Tomography; DTN, Door-to-Needle; DTCT, Door-to-CT; tPA, Tissue Plasminogen Activator (Green: EMS Pathway Specific, Yellow: Private Vehicle Pathway Specific, Blue: Both Pathways).

**Figure 2:**
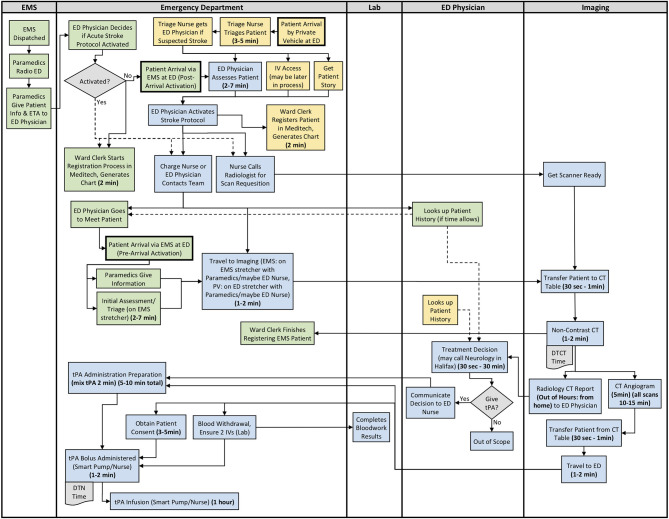
Site 2 (Rural) All Care Pathways Regular and Out of Hours Process Map. EMS, Emergency Medical Services; PV, Private Vehicle; ED, Emergency Department; CT, Computed Tomography; DTN, Door-to-Needle; DTCT, Door-to-CT; tPA, Tissue Plasminogen Activator (Green: EMS Pathway Specific, Yellow: Private Vehicle Pathway Specific, Blue: Both Pathways).

**Figure 3:**
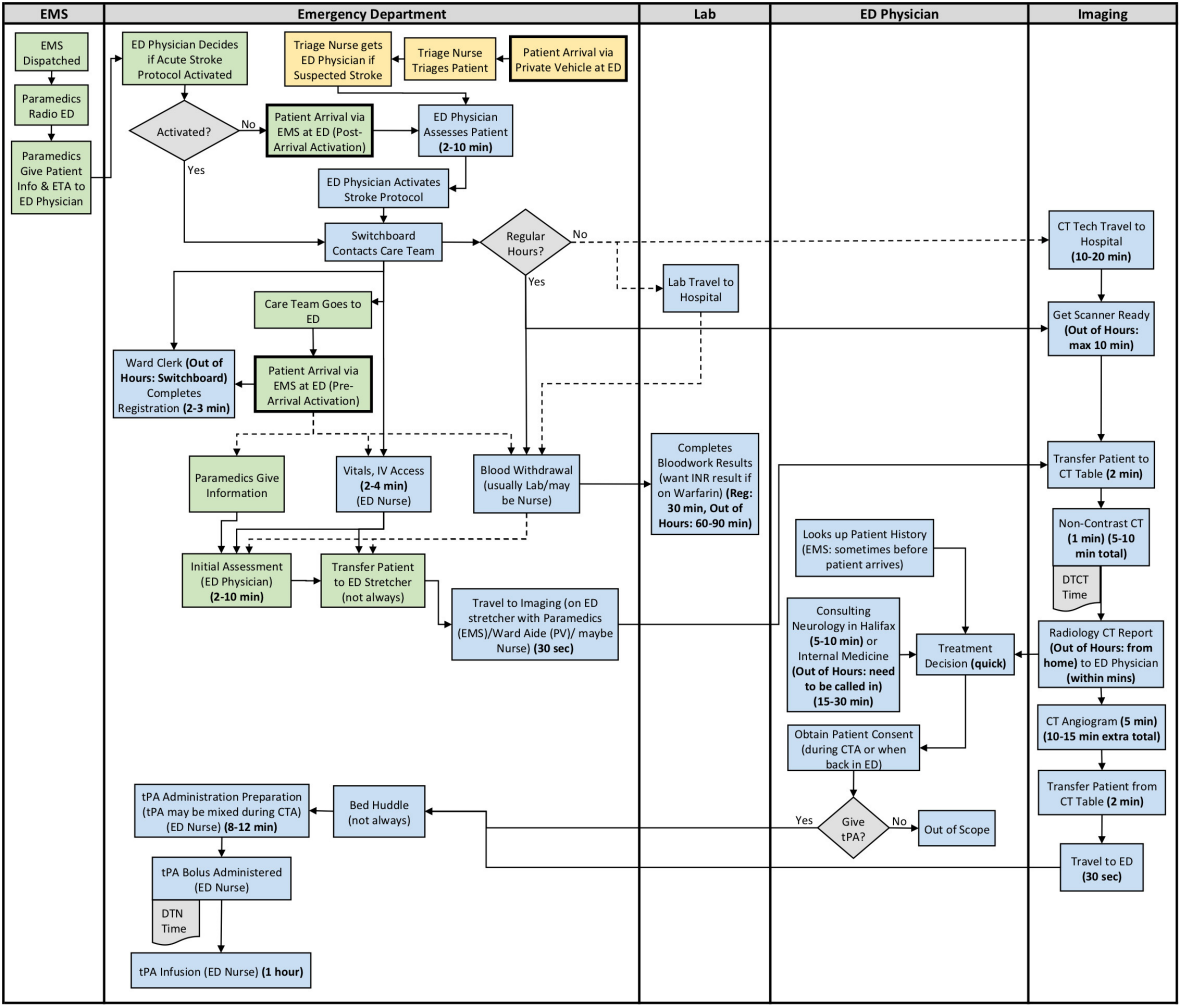
Site 3 (Rural) All Care Pathways Regular and Out of Hours Process Map. EMS, Emergency Medical Services; PV, Private Vehicle; ED, Emergency Department; CT, Computed Tomography; DTN, Door-to-Needle; DTCT, Door-to-CT; tPA, Tissue Plasminogen Activator (Green: EMS Pathway Specific, Yellow: Private Vehicle Pathway Specific, Blue: Both Pathways).

The results from section 2 of the interview guide were used to develop detailed process maps. A process map illustrating both the EMS and private vehicle pathways was developed including regular and out of hours for each site. The process maps developed for Site 1, Site 2, and Site 3 are shown in Figures 1–3, respectively. Activity durations are shown in bold within the process maps, while resources associated with certain activities are shown in brackets. Activities shown in green are unique to the EMS pathway, activities shown in yellow reference the private vehicle pathway, and blue activities show activities occurring in both pathways. The main healthcare professionals involved at each site are shown in Table 8. It is clear that there are more healthcare professionals involved at the urban site when compared to the rural sites. It is important to note that the EMS and arrival by private vehicle treatment pathways are identical once the patient is at imaging.

**Table 8:** Main healthcare professionals involved during regular or out of hours.

**Site**	**Main Healthcare Professionals Involved during Regular or Out of Hours**
Site 1 (Urban)	Paramedics (via EMS), triage nurse (arrival via private vehicle), data processing clerk, ED nurses, acute stroke nurse, ED physician, neurology residents, staff neurologist, CT technologist, and neuroradiologist
Site 2 (Rural)	Paramedics (via EMS), triage nurse (arrival via private vehicle), ward clerk, ED nurses, ED physician, CT technologist, and radiologist
Site 3 (Rural)	Paramedics (via EMS), triage nurse (arrival via private vehicle), ward clerk, ED nurses, ED physician, CT technologist, and radiologist

*EMS, Emergency Medical Services; ED, Emergency Department; CT, Computed Tomography*.

The major treatment process steps that are included at all of the sites are the following. Note that each hospital has a code stroke protocol, but the sequence of the activities and the healthcare professional responsible for the activities may differ at each hospital: (1) potential stroke (Cincinnati stroke screen positive from EMS). (2) patient registration; (3) getting IV access (thrombolysis requires 2 IVs); (4) bloodwork (drawing a bloodwork sample before administering tPA); (5) physician assessment (stroke diagnosis, determination of type of stroke syndrome, stroke severity, and eligibility for thrombolysis treatment); (6) imaging; (7) tPA bolus administration (initial 10% of medication the patient will receive); and (8) tPA infusion administration (remaining 90% of medication). Note that imaging at all sites includes a non-contrast CT head to spot a hemorrhage and assess ischemic changes (25), which is critical in determining tPA eligibility, as well as a CT Angiography (CTA) of the head and neck to assess collaterals and determines their eligibility for further EVT treatment (26). Site 1 additionally completes a CT Perfusion that measures cerebral blood flow, blood volume, and mean transit time.

Resource differences to note during out of hours are the following:

*All Sites:* Radiologist (neuroradiologist at Site 1) views and interprets the images from home and communicates with the physician.

*Site 1:* Stroke nurse unavailable on weekends and after 5 pm, Monday to Friday (Site 1 currently trying to secure this position to be available at all times). Out of hours, tPA is administered back in the ED, as opposed to in the CT department outside of the scanning area with the stroke nurse.

*Site 3:* Monday to Friday, CT technologist only scheduled for ~50% of 4 pm to 12 am shifts. On weekends, CT technologist only scheduled for ~50% of 8 am to 4 pm, and 4 pm to 12 am shifts. There is no CT technologist in-hospital from 12 am to 8 am on any day, or ward clerk from 11 pm to 7 am. Generally must call in Lab staff and Internal Medicine out of hours.

Site process details that may have contributed to differential DTN times are described in Table 9, with many illustrated in the developed process maps in Figures 1–3. The differences among sites include: protocol clarity, use of parallel processing and order of activities, transport and distance to imaging, CT technologist availability, and treatment decision consultation.

**Table 9:** **Site process details that may have contributed to differential door-to-needle times.**.

**Process Detail**	**Site 1 (Urban)**	**Site 2 (Rural)**	**Site 3 (Rural)**
Protocol clarity	Well-known by healthcare professionals.	Well-known by healthcare professionals	Majority of protocols well-known, imaging protocol requires clarification. Some physician variability in process
Patient arrival	Nurses and physicians working in parallel.	Nurses and physicians working in parallel	Nurses and physician working sequentially
Bloodwork	Collected before imaging. Have INR point-of-care machine	Collected after imaging	Collected before imaging
Transport to imaging	Remains on EMS stretcher.	Remains on EMS stretcher.	Often transferred to ED stretcher.
Distance between ED and imaging	Approx. 2–5 min	Approx. 1–2 min	Approx. 30 s
CT technologist availability	Always available	Always available	Available during regular hours, some evenings/weekend shifts. May need to travel to hospital
Treatment decision consultation	Not required	May want consultation from Site 1, physician dependent	Wanting consultation from local internist or Site 1
When tPA is being mixed	In parallel with patient in imaging	In parallel with patient in imaging	Generally after imaging, sometimes in parallel with imaging, physician dependent
tPA administration location	Outside of imaging area in regular hours with stroke nurse, in ED out of hours	ED	ED

INR, International Normalized Ratio; EMS, Emergency Medical Services; ED, Emergency Department; CT, Computed Tomography; tPA, Tissue Plasminogen Activator.

## Discussion

This qualitative study has highlighted that although the thrombolysis process in urban and rural settings have many processes in common, there are process differences as well as differences in how thrombolysis of AIS patients is viewed in these settings. The main differences stem from healthcare professionals' expertise, resource availability, and frequency of treating AIS patients. In a rural setting, an emergency physician rather than a neurologist is making the decision to treat AIS patients with tPA, which presents some disparity in how AIS patients are treated in these rural settings. Additionally, this study shows that access to rapid imaging interpretation from radiology is also a barrier. This urban-rural disparity in stroke treatment is consistent with previous quantitative studies that showed that rural AIS patients are less likely to receive thrombolysis (27) and have longer DTN times (14) than their urban counterparts. This disparity is apparent despite the use of tPA in AIS patients being reported as successful in rural settings, or in areas where access to a neurologist and imaging expertise is limited (28).

This study showed that while some Nova Scotian ED physicians felt confident in treating patients with tPA, some rural ED physicians had reservations about tPA, they indicated that the evidence was lacking leading to discomfort with treating AIS patients. This sentiment was echoed in a study conducted in 2010 in Michigan measuring the attitudes and beliefs of ED physicians, as only 49% of ED physicians surveyed indicated that science regarding the use of tPA in stroke is convincing (29). Another area that has different views among physicians is informed consent for thrombolysis, which can delay treatment, which is consistent with other studies (30, 31). However, better more streamlined communication when obtaining consent for thrombolysis can improve treatment times (31).

These results highlighted that the infrequency of treating AIS patients with tPA at rural hospitals is another factor contributing to longer DTN times. The rural sites noted that it is not uncommon to treat only 5 to 10 AIS patients annually with tPA, which can lead to lower confidence and comfort with providing the treatment, as well as less familiarity with the care pathway, which can lead to longer DTN times. Providing emergency physicians with support in treatment decision from a neurologist can assist with this deficiency. The desire for decision support for tPA was also found in the Michigan study mentioned above, as 65% of ED physicians surveyed indicated they would be uncomfortable using tPA without a consultation, but assuming the ideal setting for tPA use existed, 83% of physicians would use tPA to treat AIS patients (29). A centralized telestroke system is one proven method to provide decision support that helps to alleviate the disparity that is apparent in rural hospitals (32–34). Telestroke can also provide support in timely interpretation of imaging to overcome lack of resource availability, such as radiology, which is faced by rural hospitals.

The development of the process maps showed that there are some differences in the thrombolysis process between urban and rural hospitals. The process maps highlight some of the resource challenges at small rural hospitals where diagnostic imaging and laboratory technologist need to travel to the hospital during non-business hours. However, there are some efficiencies as well, there are more professionals involved in the urban hospital, which results in more steps prior to the patient being transferred to the CT scanner. Similarly, the time travel to the scanner is often shorter at rural hospitals, and it can be easier to obtain the necessary drugs. These process maps show that through further improvements at rural hospitals, shorter DTN times are possible. This includes pre-notifying the imaging technologists of an incoming code stroke patient when the paramedics provide pre-notification, which would allow the CT technologist enough time to travel to the hospital and prepare for the patients arrival. These would help to reduce system delays (9), while telestroke or other decision support processes can help to increase comfort with the use of tPA on AIS patients.

### Limitations and Future Direction of Research

A limitation of the study is only three hospitals were studied in a single Canadian province. However, the challenges described above such as lack of comfort with tPA treatment and lack of resources is not unique, as similar challenges have been found in other jurisdictions (29–31). Another study limitation is the small sample size of participants involved at each site, and that only two rural sites were included. Due to the number of participants, only preliminary conclusions can be drawn as there were not a sufficient number of participants to remove individual biases. Additionally, not all professionals that are part of the treatment process were involved in the interview process at each site; the current study primarily excludes paramedics and radiologists. There were two rural sites in the study that were felt to represent the province adequately, but for further accuracy regarding the urban-rural treatment differences additional rural hospitals should be considered. Furthermore, this study was limited to thrombolysis, and endovascular treatment (EVT) was not included. We recognize that rural hospitals face additional challenges in arranging transfer of patients for EVT, which deserves further study.

Study next steps include the development of a discrete-event computer simulation model using the process maps that were developed. The purpose of the simulation is to be able to run scenarios for various changes, which will quantify the impact of changes through a modeled reduction in DTN times. Examples of process changes to be included in the simulation include: always administering tPA outside of the imaging area (10, 35–37), obtaining the patient's blood sample in parallel with other arrival activities before going to imaging, transporting the patient on the EMS stretcher to imaging (10, 11, 35, 36, 38), pre-notifying imaging technologists of an arriving stroke, and further use of parallel processing (36). Simulation modeling was determined to be the most applicable tool for the study as it lends well to the overall goals in terms of assessing and modifying treatment paths. Simulation-based approaches are effective in assessing solutions in the stroke pathway (39), and can successfully be used as a decision-making tool before committing real resources (40).

## Conclusion

This qualitative study with clinicians involved in the tPA treatment of AIS patients at efficient and inefficient rural hospitals and at an urban hospital revealed the disparities between urban-rural hospitals in the treatment of AIS patients with tPA. Some of the key disparities between urban-rural hospitals are rooted in emergency physician's being the treating physician at rural hospitals, as many are not comfortable with treating with tPA and often treat infrequently. Additionally, the developed process maps visually highlighted streamlined portions of the treatment pathway for each site, as well as inefficiencies to be addressed. The majority of treatment delays encountered are system delays, which can be appropriately planned for in order to reduce delays within the care pathway. There is a general consensus of an urban-rural treatment gap for AIS patients in Nova Scotia, and that continuing education is key to improving ED physician comfort in regard to the evidence surrounding tPA and the thrombolysis treatment decision when treating patients with tPA in rural hospitals. Thrombolysis service alternatives, such as telestroke, could help in reducing the urban-rural treatment gap and improve ED physician comfort with treating with tPA. Study next steps include the development of a discrete-event computer simulation model to quantify the impact of these suggested changes to the current processes.

## Data Availability Statement

The datasets presented in this article are not readily available because of the anonymized data to protect the privacy of the individuals involved in the study. Requests to access the datasets should be directed to the corresponding author.

## Ethics Statement

The studies involving human participants were reviewed and approved by the Nova Scotia Health Research Ethics Board. The participants provided their written informed consent to participate in this study.

## Author Contributions

TB: study design, data collection, data analysis, preparation of figures and tables, and preparation of the first and revised drafts of the manuscript, as well as final editing and formatting. NK: study design, editing, formatting, and revision of manuscript for intellectual content. DV: input into study design and revision of manuscript for intellectual content. All authors contributed to the article and approved the submitted version.

## Conflict of Interest

The authors declare that the research was conducted in the absence of any commercial or financial relationships that could be construed as a potential conflict of interest.
