# Supplementary material for: Analysis of Thrombolysis Process for Acute Ischemic Stroke in Urban and Rural Hospitals in Nova Scotia Canada
Source: Front Neurol. 2021 Mar 15;12:645228. doi: 10.3389/fneur.2021.645228 (PMC8005571; doi:10.3389/fneur.2021.645228)
Supplement: Supplementary file 1 [file Data_Sheet_1.PDF]

Interview Guide for Stroke Coordinators

Introduction

I will ask you a series of questions aimed to gain a detailed understanding of the process for the treatment of acute ischemic stroke (AIS) patients with alteplase, otherwise referred to as tPA (tissue plasminogen activator), at your facility and your role in the process. The questions included are considered an interview guide, as you are encouraged to add comments that are seen to be beneficial to the topic.

Participant Information

1. What is your profession and role at this hospital? (ie: ED physician, paramedic, nurse, etc.)
2. If follow-up questions are required during the project life, are you open to be interviewed again?

Section 1

Contextual Information

3. What is considered an appropriate time frame for treating a patient with tPA?
    - i.e.: Within 3 hours, 4.5 hours, etc.
4. What priority do acute (within 3 hours of onset) ischemic stroke patients receive at your hospital?
5. How many of each health professional resources are involved, in regular and off hours, with treating acute ischemic stroke patients?
6. Are there human resource limitations at your site? Example: is it a challenge to retain certain positions at this location.
7. Are there issues with availability of tPA for thrombolysis treatment?
8. What are the key metrics you review for this process when completing data analysis?
9. Do you feel there is a gap in acute ischemic stroke treatment between urban and rural hospitals?
10. Would you consider the acute stroke treatment process to be effective?
11. Would you consider the acute stroke treatment process to be time efficient?
12. Do you feel protocols are always followed?
13. What improvements can you think of that would increase treatment process efficiency at your site?

Treatment Delay Factors

14. What patient-related factors do you feel are the most common at your site?
    - Examples: delays due to management of hypertension, delays due to management of emergent medical condition, delays due to unclear time of onset, etc.
15. What system factors do you feel are the most common at your site?
    - Examples: delays due to stroke diagnosis, delays due to obtaining CT, delays due to obtaining laboratory results, etc.
16. Are patient-related factors or system factors the main source of treatment delays at your site?

Section 2

Treatment Process

17. Please describe the acute stroke treatment process.
    - The objective is to gather information to develop a process map of the treatment process at your site.
18. What are considered regular hours at your site?
19. Does your acute stroke treatment process differ during regular hours or any other hours?
20. Does your acute stroke treatment process differ if the patient arrives by ambulance?
21. Does EMS pre-notify this hospital of an incoming stroke patient?
22. How does patient registration work at your site?
    - Does this process differ if the patient arrives by private vehicle or EMS?
23. How do patients arrive at the CT scanner?
    - How many scanners are there?
24. Who makes the decision that a patient will receive tPA?
25. Where does the tPA get administered in the hospital?
    - Where is tPA stored?

Additional Comments

If there are any further details or comments that were not covered in the included questions above, please share what you feel will be beneficial to the understanding of the acute ischemic stroke treatment process, and the desired improvements to be implemented.
