# Supplementary material for: Analysis of Thrombolysis Process for Acute Ischemic Stroke in Urban and Rural Hospitals in Nova Scotia Canada
Source: Front Neurol. 2021 Mar 15;12:645228. doi: 10.3389/fneur.2021.645228 (PMC8005571; doi:10.3389/fneur.2021.645228)
Supplement: Supplementary file 2 [file Data_Sheet_2.PDF]

Standard Interview Guide

Introduction

I will ask you a series of questions aimed to gain a detailed understanding of the process for the treatment of acute ischemic stroke (AIS) patients with alteplase, otherwise referred to as tPA (tissue plasminogen activator), at your facility and your role in the process. The questions included are considered an interview guide, as you are encouraged to add comments that are seen to be beneficial to the topic.

Participant Information

1. What is your profession and role at this hospital? (ie: ED physician, paramedic, nurse, etc.)
2. If follow-up questions are required during the project life, are you open to be interviewed again?

Section 1

Contextual Information

3. Do you feel comfortable treating acute ischemic stroke patients?
4. What are your perceptions about treating acute ischemic stroke patients with tPA?
5. What do you consider an appropriate time frame for treating a patient with tPA?
    - i.e.: Within 3 hours, 4.5 hours, etc.
6. What priority do acute (within 3 hours of onset) ischemic stroke patients receive at your hospital?
7. What other professions are included in the acute stroke process at your site?
8. Are there issues with availability of tPA for thrombolysis treatment?
9. Is patient consent an issue when conducting thrombolysis treatment with acute ischemic stroke patients?
10. Do you feel there is a gap in acute ischemic stroke treatment between urban and rural hospitals?
11. Would you consider the acute stroke treatment process to be effective?
12. Would you consider the acute stroke treatment process to be time efficient?
13. What improvements can you think of that would increase treatment process efficiency at your site?

Treatment Delay Factors

14. What patient-related factors do you feel are the most common at your site?
    - Examples: delays due to management of hypertension, delays due to management of emergent medical condition, delays due to unclear time of onset, etc.
15. What system factors do you feel are the most common at your site?
    - Examples: delays due to stroke diagnosis, delays due to obtaining CT, delays due to obtaining laboratory results, etc.
16. Are patient-related factors or system factors the main source of treatment delays at your site?

Section 2

Treatment Process

17. Please describe the acute stroke treatment process.
    - The objective is to gather information to develop a process map of the treatment process at your site.
18. Describe your role in the process and the durations of the included activities.
19. What are considered regular hours at your site?
20. Does your acute stroke treatment process differ during regular hours or any other hours?
21. Does your acute stroke treatment process differ if the patient arrives by ambulance?
22. Does EMS pre-notify this hospital of an incoming stroke patient?
    - If yes, please describe what happens when your hospital receives the notification.
23. What happens when the patient first arrives at the hospital?

24. How does patient registration work at your site?
    - Does this process differ if the patient arrives by private vehicle or EMS?
25. Is blood work completed on all patients?
26. Are creatinine blood tests performed on patients?
27. Do you wait for the INR (International Normalized Ratio) results before administering tPA?
28. How do patients arrive at the CT scanner?
    - How many scanners are there?
29. Who makes the decision that a patient will receive tPA?
30. Who mixes the tPA and when does this occur?
    - Where is tPA stored?
31. Is a CTA (Computed Tomography Angiography) scan completed for patients who will be receiving tPA?
    - If yes, when is the CTA scan completed within the treatment process?
32. Who administers the tPA?
33. Where does the tPA get administered in the hospital?

Additional Comments

If there are any further details or comments that were not covered in the included questions above, please share what you feel will be beneficial to the understanding of the acute ischemic stroke treatment process, and the desired improvements to be implemented.
